# Misalignment of social support in the social media posts of young adult cancer caregivers

**DOI:** 10.1002/cnr2.1998

**Published:** 2024-02-22

**Authors:** Sara G. Bybee, Austin R. Waters, Keely Smith, Echo L. Warner

**Affiliations:** ^1^ College of Nursing, University of Utah Salt Lake City Utah USA; ^2^ Huntsman Cancer Institute at the University of Utah Salt Lake City Utah USA; ^3^ The University of Arizona, College of Nursing Tucson Arizona USA

**Keywords:** cancer, caregiving, mental health, psycho‐oncology, social support, young adult

## Abstract

**Background:**

Compared with older cancer caregivers, young adult cancer caregivers (YACC) experience greater stress and depression during the first 6 months of caregiving. Social support can buffer the negative psychological effects associated with cancer caregiving. However, the misalignment of social support is associated with increased distress and worse emotional well‐being. As YACC are more likely to utilize social media (SM) to seek social support compared with older cancer caregivers, they may be at greater risk of experiencing the misalignment of social support and related negative outcomes.

**Objective:**

The purpose of this study was to identify ways that SM posts containing support for YACC's were potentially misaligned with the social support desired by YACC.

**Methods:**

In this secondary analysis, cancer‐related SM posts (*N* = 760) from 34 YACC's SM accounts were extracted for 6 months following the day of cancer diagnosis and imported into NVivo qualitative analysis software. Open coding of posts from four YACC identified a pattern of SM posts containing responses that may misalign with social support requests, and could be perceived as dismissive of YACC's emotions. Similar posts were grouped together, forming categories which were used to create a codebook and applied in coding all posts from the remaining 30 YACC.

**Results:**

Content analysis identified three categories of social support misalignment originating from YACC's SM posts: supplications (*n* = 251, 33%), prevailing (*n* = 7, 10.1%), and consolations (*n* = 16, 2.1%). Supplications involved prayer or trust in god, prevailing posts compared dealing with cancer to a fight and suggested that the person would overcome cancer, and consolations used quotes, song lyrics, and or general reassurances in SM posts.

**Conclusions:**

Findings suggest that when YACC use SM during cancer experiences, they may interpret SM posts as social support or as misalignment of social support requests, which in turn can lead to either improved quality of life or additional distress (respectively). This study adds to the body of knowledge regarding how YACC use social media for social support and may inform future interventions designed to match YACC's desired support with support offered.

## 1 INTRODUCTION

### 1.1 Background

In 2015, approximately 1.46 million young adults were caregivers for someone with cancer.^1^ Providing care to an individual with cancer versus another disease is associated with a greater number of caregiving hours, and a greater likelihood of assisting with instrumental or other activities of daily living.^2^ Young adults often assume the role of cancer caregiver unexpectedly and balance these new responsibilities with others involved in the transition to adulthood including completing education, establishing careers, dating, and family formation.^3^, ^4^ Possibly due to these competing demands, compared to older cancer caregivers, young adult cancer caregivers (YACC)—defined as adults between the ages of 18 and 39^5^, ^6^, ^7^, ^8^—experience more stress and depression during the first 6 months of caregiving.^9^

Young adults, including YACC, also utilize social media (SM) at much higher rates than older populations. In 2021, 84% of adults ages 18–29 and 81% of adults 30–49 reported ever using SM compared with only 45% of those 65 and older.^10^ YACC may use SM to garner social support, stay connected with family or friends, and share updates about the cancer patient.^5^, ^11^, ^12^ Research demonstrates that social support has protective effects on health, including for cancer caregivers.^13^, ^14^ Thus, engaging with online networks for social support may help reduce the burden experienced by YACC.^13^

While the benefits of social support are ill‐refuted, those who attempt to provide support to YACC may unintentionally engage in what is popularly known as toxic positivity—the excessive and ineffective overgeneralization of a positive state even in the worst circumstances.^15^, ^16^ Toxic positivity involves someone stating “It could be worse” or “Stay positive” when responding to an individual encountering a legitimately difficult time.^17^ Toxic positivity can also manifest as social support misalignment—when an individual requests or expects one kind of support and is offered another type.^18^, ^19^ While SM allows for the rapid dissemination of information with others, SM may provide users with a false sense of anonymity or with increased distance between users which can lead to miscommunication or misinterpretation.^20^ Thus, as YACC utilize SM with greater frequency than older caregivers and are often utilizing SM to elicit social support, they may have a greater risk of experiencing the misalignment of social support and its negative outcomes.

Social support misalignment can occur when the “timing, type of support, desire for support, or the nature of delivery are not matched to existing needs.”^18^ For example, a cancer caregiver may request help with providing transportation for the cancer patient (i.e., instrumental support) and misalignment occurs when this type of request is met with emotional support (e.g., “I know you can find them a ride!”) Prior research has demonstrated that unsupportive social interactions may have a large effect on psychological outcomes as these interactions can deny, minimize, and invalidate individuals' experiences.^21^

One study involving individuals with breast cancer found that the most frequent unsupportive and unhelpful responses were those in which an individual told the individual with cancer that they should be “strong” and “keep their chin up.” Other unhelpful and unsupportive responses included comments that attempted to minimize the situation or force optimism.^21^ In sum, research demonstrates that the misalignment of social support is associated with greater social disruption, worse emotional well‐being,^21^ poor psychosocial adjustment to cancer,^19^ symptoms of anxiety, and depression.^18^

On the other hand, social support alignment, or what is referred to in the literature as optimal matching theory or goal‐matching, suggests that “if the proper type of support is provided in a particular context, there will be an enhancement of quality of life and adjustment to the disease and its treatments compared with a situation in which the need and the provision of support are mismatched.”^22^ Optimally, social support perceived by YACC is matched to the type and timing desired by the YACC which will then enhance their quality of life. Despite evidence demonstrating the benefits of social support as well as evidence of the potential unintended consequences of the misalignment of social support, few peer‐reviewed studies explore the misalignment of social support using SM, nor do they focus on YACC.

### 1.2 Study purpose

With the widespread use of SM among young adults and YACC's use of SM to garner social support, it is critical to understand whether and how discourse could be harmful or helpful to the health and wellbeing of YACC. To address this gap, the purpose of this exploratory study was to identify ways that SM posts containing support for YACCs were potentially misaligned with the social support desired by YACC.

## 2 METHODS

### 2.1 Participants and recruitment

This study was part of a larger mixed‐methods study conducted with YACC about their use of SM for social support and changes in support types over the first 6 months of caregiving experiences.^5^, ^6^, ^7^, ^8^ YACC were eligible for this study if they were 18–39 years old, spoke English, used SM weekly, and had been serving as a caregiver to someone with cancer for at least 6 months. Stratified purposive sampling was used to recruit eligible YACC; Participants were recruited via flyers and in‐person at a National Cancer Institute (NCI)‐designated comprehensive cancer center in the Intermountain West. SM advertisements and referrals from cancer patients diagnosed 6 months to 5 years prior were also used to recruit participants. A total of 354 individuals with cancer were screened for study eligibility, which resulted in 61 potential caregiver participants. Thirty‐four YACC enrolled in the study, completed informed consent, a brief survey and telephone conversation, and gave their consent for investigators to access their social media accounts (Facebook, Instagram, and Twitter) from the time of the patient's diagnosis to 6‐months‐post diagnosis.

### 2.2 Data collection

#### 2.2.1 Sociodemographic and cancer patient factors

The following sociodemographic variables were collected from YACC through close‐ended questions asked via telephone: age, gender, race, ethnicity, employment status, educational attainment, and if they were caring for anyone besides the cancer patient. Cancer patients' age, relationship with YACC, and time since their cancer diagnosis were also collected.

#### 2.2.2 Characteristics of SM posts

All posts and responses in the 6‐months following the cancer patient's diagnosis were extracted from YACC's SM by authors ELW, ARW and research assistant TN (see acknowledgments). After a brief telephone conversation, 6‐months of SM data were extracted retrospectively from participants' SM accounts. This data collection occurred over a 9‐month period from January to October, 2018. Since cancer patients were diagnosed on differing dates, the 6‐month period of extracted data post‐diagnosis was different for each YACC. Data extracted from YACC's SM included: any text posted, presence of any visual content such as photos or video, the total number of likes, comments and shares for each original post, the number of characters, word count, type of SM platform (Instagram, Facebook, or Twitter), and the date of the post.

#### 2.2.3 Content analysis of SM posts

SM posts (*N* = 2322) written by YACC and comments in response to that particular participant (over a 6‐month period) were manually extracted and combined into one Microsoft Word document so that there was one document for every YACC participant. All word documents from *N* = 34 YACC were imported into NVivo 12 software for qualitative analysis.^23^ All posts with cancer‐related content (*N* = 777) were imported into a Microsoft Excel spreadsheet so each row contained one post or one response to an original post.

### 2.3 Data analysis

#### 2.3.1 Sociodemographic and cancer patient factors

Descriptive statistics were calculated for YACC sociodemographic and cancer patient factors using Stata 14.2.^24^ This included means, standard deviations, and frequency distributions.

#### 2.3.2 Characteristics of SM posts

SM variables were analyzed using Excel by calculating descriptive statistics including frequency distributions and means.

#### 2.3.3 Content analysis of SM posts

The first step in the analysis of the SM posts was to exclude posts that did not contain cancer‐related content. Qualitative analysis of SM posts related to cancer began with open coding, in which patterns and initial reactions were noted.^25^ The following steps were taken by author SB: (1) open coding techniques were used to examine at least 10% of YACC's SM data (posts from *N* = 4 YACC, 11.8%)^26^; (2) posts with similar messaging, content, and the potential for the misalignment of social support were then grouped together; (3) each group of similar posts was examined for commonalities. The research team discussed the three categories that emerged from the analysis and how these differed from general social support categories (e.g., emotional, informational, validation, companionship, and instrumental^5^), and came to consensus on initial code labels and definitions. SB drafted the codebook which contained code labels, definitions, and exemplar quotations, which was then used by SB to code all remaining posts.^27^ In developing the codebook, online searches of keywords (e.g., prayer, healing from God, beating illness/cancer, placate, and assure/reassure) helped to define and name the three categories reflecting the misalignment of social support.

The three categories of the misalignment of social support identified in this analysis were supplications, prevailing, and consolations, which often presented as being misaligned with support requests made by the YACC. For example, posts containing supplications involved responses about communicating with a deity, including prayer or a call for help from God and also contained references to a deity providing peace, blessings, and or healing to YACC or the individual with cancer. Prevailing SM posts contained discourse about the patient's cancer being triumphed over due to the patient's strength or superiority and or contained references to battle, war, combat, fighting, and other conflict, regardless of the likelihood the patient would survive or succumb to their cancer. Consolations were defined as quotations, song lyrics, and or general statements of reassurance in response to YACC statements of uncertainty (see Table 1 for code labels, definitions, and exemplar quotations).

**TABLE 1:** Definition and examples of the misalignment of social support.

Code label	Definition	Exemplar quotes
Supplications	Posts describing a form of communicating with a deity, such as prayer or a call for help from God; includes references to a deity providing peace, blessings, healing to YACC or the individual with cancer.	“My prayers are with you.” “We pray for you to feel peace.” “Thanks to God, everything will be ok.” “The lord will bless you.”
Prevailing	When posts talk about the patient's cancer being triumphed over due to the patient's strength or superiority; this may include references to battle, war, combat, fighting, and so forth.	“You're going to beat this.” “Kick cancer's butt!” “Having an army behind you helps the fight.” “This is a battle you can win.”
Consolations	Posts containing quotations, song lyrics, and or general statements of reassurance.	“Everything will be alright.” “You WILL get through this.” “We know all will be well.” “Trials are there to make us stronger.”

## 3 RESULTS

### 3.1 Sociodemographic and cancer‐related factors

A total of *N* = 34 participants participated in this study. Participants were in the following age groups: 18–24 years (*n* = 4, 11.8%), 25–29 years (*n* = 13, 38.2%), 30–34 years (*n* = 9, 26.5%), and 35–39 years (*n* = 8, 23.5%) (see Table 2 for sociodemographic and cancer‐related factors). The study sample consisted primarily of female caregivers (*n* = 24, 70.6%). Caregivers were non‐Hispanic White (*n* = 31, 91.2%) and employed (*n* = 29, 85.3%). Over half of caregivers were college graduates (*n* = 18, 53.0%) and were also caring for a child under age 18 (*n* = 21, 61.8%). The mean age of cancer patients was 37 years (SD = 13.8, range 19–76). Cancer patients tended to be spouses/partners (*n* = 18, 52.9%) or parents (*n* = 8, 23.5%), and 82.4% (*n* = 28) were diagnosed between 6 months to less than 2 years prior. Cancer patients were diagnosed with leukemia or lymphoma (*n* = 12, 35.3%), breast (*n* = 6, 17.6%) or another cancer (*n* = 16, 47.1%).

**TABLE 2:** Sociodemographic and cancer‐related factors.

Age at interview (years)	*M* = 29.0	SD = 4.72
	*N*	%
Gender		
Female	24	70.6
Male	10	29.4
Ethnicity		
Non‐Hispanic White	31	91.2
Hispanic/Latino	3	8.8
Race^a^		
White	31	91.2
Black/African American	1	2.9
Other race	5	14.7
Employment status		
Employed	29	85.3
Educational status		
College graduate or more	18	53.0
Some college	13	38.2
High school or less	3	8.8
Relationship to patient		
Spouse/partner	18	52.9
Child	8	23.5
Sibling	5	14.7
Other (e.g., niece/nephew, parent)	3	8.8
Caring for others besides patient		
Child under 18	21	61.8
Other (adult child, parent, etc.)	4	11.7

Cancer patient's age (years)	*M* = 37.0	SD = 13.8
Time since cancer diagnosis		
6 months–1 year	12	35.3
1–2 years	16	47.1
2–5 years	6	17.7

^a^ The percentages add up to greater than 100% as participants could select more than one race.

### 3.2 Characteristics of SM posts

**FIGURE 1:**
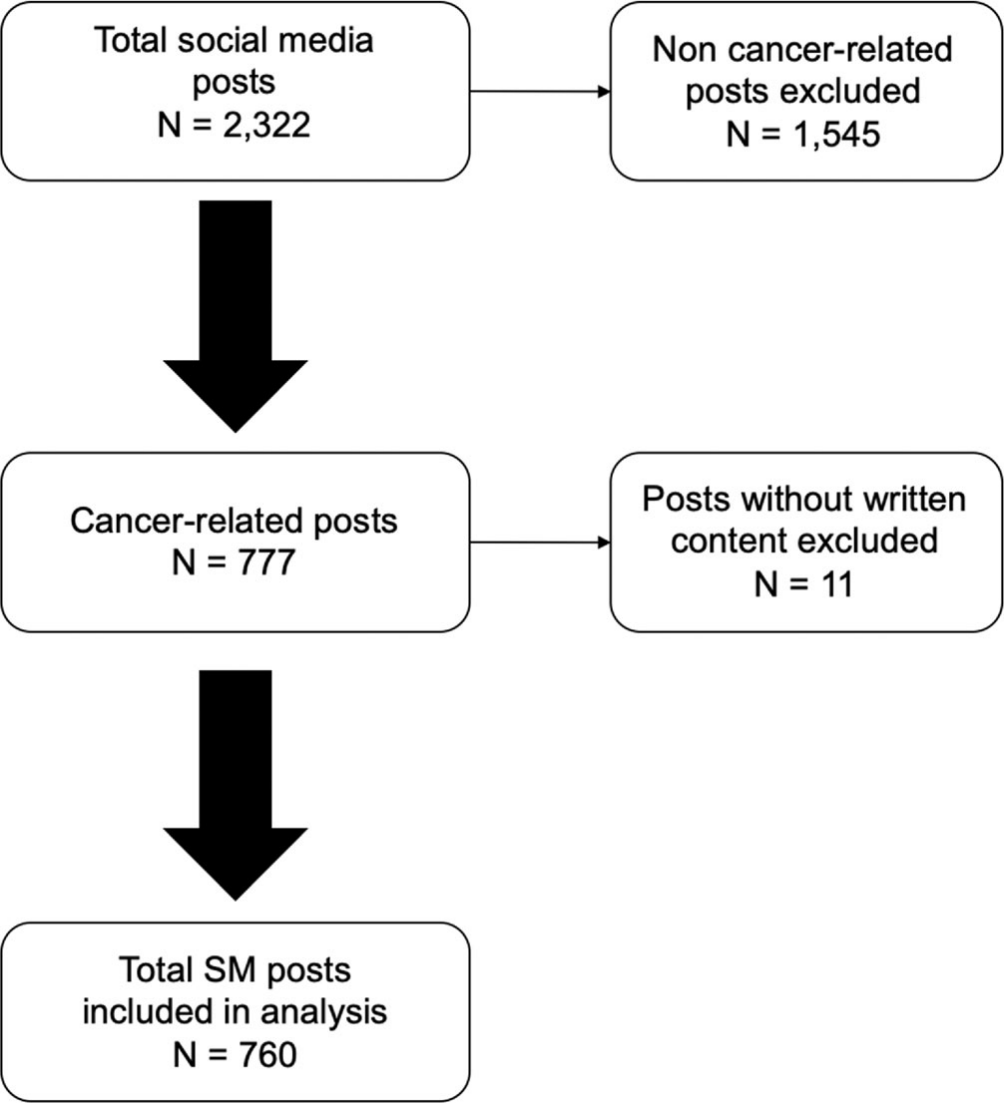
Flowchart of posts included in the analysis.

There were a total of 2322 posts extracted from YACC's SM accounts. Of these posts, 777 (33.4%) contained cancer‐related content and were therefore included in the analysis (see Figure 1 for a flowchart depicting the inclusion of SM posts).

Eleven posts were excluded due to the lack of written content, resulting in a total of 760 posts included for analysis. Of the 760, 186 (24.5%) were written by the YACC participant and 38 (20.4%) of these were original posts, meaning that the YACC posted the content as opposed to a response (a comment) on a post. Out of the 760 cancer‐related posts that contained written content, 574 (75.5%) were written by someone other than the participant and 51 (6.7%) posts contained original content; the remaining (709, 93.3%) were comments to these original posts. The majority of posts (*n* = 434, 57%) contained no photographs.

### 3.3 Content analysis of SM posts

Utilizing the codebook, 344 (45.3%) posts fell into one of the three categories of social support misalignment. There were 251 (33%) posts containing supplications, 77 (10.1%) posts containing prevailing, and 16 (2.1%) with consolations. There was some overlap in the types of misalignment present in each post. For example, there were 23 posts that contained both supplications and prevailing, four posts with supplications and consolations, and one post with supplications, prevailing, and consolations. There were no posts that contained only prevailing and consolations. The remaining 416 (54.7%) cancer‐related posts and comments to posts which were not categorized as misalignments of social support contained messages that could not be perceived as a misalignment of social support. For example, some of these posts contained responses such as “thank you for keeping us updated” that were not support (misaligned or otherwise).

## 4 DISCUSSION

### 4.1 Principal results

The SM accounts of YACC demonstrate the possibility for the misalignment of social support including supplications, prevailing, and consolations. To our knowledge, as there are no prior studies examining the potential for SM posts to be perceived as misalignments of social support, we cannot make direct comparisons between this exploratory study and prior work. However, our identification and categorization of misalignment of social support seems to be supported by prior research conducted in the areas of toxic positivity, optimal matching, and cancer caregiving.

Similar to our category of *prevailing*, cancer patients have reported facing restrictive discourse “including war and sport metaphors espousing the ‘fighting of cancer’ and ‘toughing it out’.”^21^, ^28^ Furthermore, among cancer survivors, the relationship between optimism and positive affect have been shown to be mediated by adopting a “fighting spirit.”^29^ Self‐healing and self‐responsibility—similar to *supplications*—for disease progression was also discussed as counterproductive by cancer patients in previous studies.^28^ Prior research with cancer caregivers also found that caregivers of patients with advanced cancer reported that their faith and religiosity helped them cope with the patient's cancer.^30^ Caregivers who were deeply spiritual or religious demonstrated lower levels of anxiety, depression, and improved quality of life.^30^

False reassurance—when you tell someone they will be okay even though you do not know they will be, which has previously been described in media on “toxic positivity,” is consistent with *consolations*.^16^ In fact, some research highlights the fact that cancer caregivers felt “consoled” by social support from family, friends, and neighbors after the death of the cancer patient.^31^ Despite the similarities to prior research conducted in the context of the misalignment of social support, cancer, and caregiving,^20^, ^28^, ^30^ it is unknown how and under what circumstances supplications, prevailing, and or consolations could negatively impact YACC's mental health, and therefore this would be a potential area for future research.

While responses to YACC's posts on SM may be intended to be supportive, responses containing any of these three categories of misalignment could have been interpreted by YACC as either insensitive and misaligned with their desired support, or as truly supportive and uplifting. The way that YACC receive these SM posts depends upon their specific circumstances, the relationship between the YACC and the individual posting, and or the posting individual's specific circumstances. For example, for a YACC who has a strong faith in God, a SM post stating, “God will heal all” may feel reassuring, while for a YACC who is agnostic, this same statement may seem dismissive of their experience as a cancer caregiver. It is important to note that SM posts containing supplications, prevailing, and consolations have the potential to invalidate YACC's emotions and invoke guilt or shame, which may result in worse psychological health.

Resorting to supplications, prevailing, and consolations in YACC's SM may reflect an unease in discussing the difficult feelings surrounding cancer in young adults, most of whom have no prior exposure to severe illness, and may serve to reassure the individual posting rather than the YACC or the individual with cancer. Young adults may lack experience with severe illness and may simply not know how to respond to someone providing care to an individual with a life‐limiting disease.^4^, ^32^

Future research could examine the overlap of the misalignment categories identified in SM posts. Supplications and prevailing were the two categories that overlapped the most with one another. The coupling of prayer and a fighting attitude is logical given that for many individuals, prayer may be seen in of itself as a way to “fight” one's battles: “Praise be to the lord my rock, who trains my hands for war, my fingers for battle.”^33^ The fact that there were no posts that contained only prevailing and consolations may suggest an inherent dichotomy between these two concepts. That is, adopting a prevailing attitude over cancer is akin to acknowledging the threat of cancer and encouraging the individual with cancer to preside over that threat. Engaging in consolations (by suggesting that everything will work out for example), denies that there is a real threat to with which to contend.

Furthermore, future research could examine the individual effects of supplications, prevailing, and consolations. In an increasingly technology‐based world where young adults are turning more and more to digital connectedness, it is vital that those connections promote wellness and social capital.^34^ As the incidence of cancer is expected to increase 49% by the year 2050, there will be increasing numbers of cancer caregivers who will likely seek support through SM and other internet‐mediated methods.^35^ If the “positive thinking” ideology persists without balance of other negative emotions, some cancer caregivers' mental health may suffer, as accepting negative emotions^36^ and sharing cancer‐related concerns with a trusted individual^37^ have been shown to be beneficial for psychological health.

The extracted posts from YACC's SM accounts were written between 2015 and 2018, before the COVID‐19 pandemic. Since the pandemic and social distancing guidelines, the use of the internet has drastically increased. In mid‐March 2020, when the coronavirus pandemic forced many Americans to work from home, internet use in America increased by 25% within a few days.^38^ This may mean that YACC may rely even more heavily upon SM for social support now than when this study was conducted. Thus, the need to understand the dichotomy of SM posts and how they can either foster or impede wellbeing is more pressing than ever.

This research raises questions regarding the benefits of using SM for seeking emotional or psychological support. SM may not be ideal for emotional support of YACC, as SM posts can be brief and require interpretation of subtle messages which can leave room for misinterpretation. Perhaps SM may be the most useful for emotional support in situations in which YACC are communicating with close friends who are aware of the YACC and patients' specific circumstances including their spirituality, religion, the cancer diagnosis, and prognosis. This way, there would be less likelihood that those posting would be inadvertently insensitive or engage in the misalignment of social support.

### 4.2 Implications for providers

Providers—including cancer care providers and mental health providers for both the individual with cancer and for YACC—can use these findings to inform their work with this population. Cancer care providers may be able to offer or connect individuals with the type of emotional and psychological support they desire. For example, for YACC who want to share and hear others' experiences with cancer caregiving, patient navigators and social workers could refer them to cancer care organizations that have support groups such as Cancer Sucks^39^ and Livestrong.^40^ YACC who prefer one‐on‐one support from a professional could be referred to therapists who provide individual counseling. As previously mentioned, by better understanding each individual's particular circumstances, providers can improve the match between desired and received support which ultimately may improve YACC's quality of life.^22^

Cancer care providers may also be able to warn YACC about the potential unintended consequences of widely using SM to seek social support. Furthermore, mental health providers can encourage young adults with cancer and YACC to share their concerns, fears, and anxieties and affirm these feelings. Acknowledging the desire to maintain an optimistic outlook while also normalizing any difficult thoughts and feelings can reassure young adults that their internal experience is normal and valid and may ultimately reduce symptoms of depression and anxiety.

### 4.3 Limitations

The study sample primarily consisted of urban non‐Hispanic White women, and therefore findings may not be generalizable beyond similar groups. In addition, this study utilized data gathered between 2017 and 2018 and may therefore not be representative of how YACC are currently using SM given the passage of time and rapid changes in SM structure and use.^5^ This study may have overrepresented individuals with spiritual and or religious affiliation (therefore producing higher numbers of supplications in SM posts) given recruitment in a geographic location where the majority of the population (67.7%) reports a religion of Latter‐Day Saint.^41^ However, this sample may also provide a unique look into how a religious population might misalign social support. We did not collect data on the stage of cancer, prognosis, or current treatment, factors which may influence social media use and the type of responses made by social networks about cancer‐related posts on SM. Given the limited knowledge of each individual posting on YACC's SM accounts, we cannot know the intention of each post, nor can we infer the response each post evoked in the YACC. Despite these limitations, to our knowledge, this is one of the first explorations of the potential for misalignment of social support in YACC's SM interactions. Findings contribute new knowledge to the fields of cancer caregiving, psychosocial support, and internet communication and research. Future research could examine the psychological outcomes of YACC interpreting posts as misalignment of social support versus YACC who experience these posts as truly supportive to determine how each message affects YACC.

## 5 CONCLUSIONS

For YACC, social support may reduce the risk of negative outcomes such as depression and anxiety. While using SM can garner social support, it also has the potential to result in miscommunication or misinterpretation. Thus, YACC seeking emotional support through SM should be prepared for the possibility that requests for social support result in the misalignment of support and the potential negative consequences of such misalignments. To limit the possibility of misalignment and increased distress, YACC could use SM to reach their larger social networks in order to coordinate instrumental support or to elicit informational support. Emotional support on the other hand, may be better obtained through close personal contacts. YACC may want to evaluate the goal of using SM in their caregiving role, and weigh the benefits and consequences of seeking particular types of social support through SM. Not only does the misalignment of social support have the potential to inform negative mental health outcomes, but “it can also be used to uphold oppression by making people ignore the oppression that is going on and encouraging them to ‘just be positive’.”^42^ By acknowledging difficult emotions and situations, we can begin to center the experiences of those struggling—whether they be difficulties with providing care to someone with cancer or the deleterious effects of discrimination—and begin addressing them in a straight‐forward, healthy manner.

## AUTHOR CONTRIBUTIONS

**Sara G. Bybee:** Conceptualization (lead); formal analysis (equal); methodology (lead); writing – original draft (lead); writing – review and editing (lead). **Austin R. Waters:** Conceptualization (supporting); data curation (supporting); writing – original draft (supporting); writing – review and editing (supporting). **Keely Smith:** Data curation (supporting); writing – original draft (supporting). **Echo L. Warner:** Conceptualization (supporting); data curation (lead); resources (lead); supervision (lead); writing – original draft (supporting); writing – review and editing (supporting).

## CONFLICT OF INTEREST STATEMENT

The authors have stated explicitly that there are no conflicts of interest in connection with this article.

## ETHICS STATEMENT

All study procedures were approved by the University of Utah Institutional Review Board (IRB #00097575) and were performed in accordance with ethical standards in the 1964 Declaration of Helsinki and its later amendments. Study participants completed informed consent and provided their permission for study team members to access to their SM feeds.

## Data Availability

The data that support the findings of this study are available on request from the corresponding author. The data are not publicly available due to privacy or ethical restrictions.
